# Community-Based Interventions for the Treatment and Management of Conflict-Related Trauma in Low-Middle Income, Conflict-Affected Countries: a Realist Review

**DOI:** 10.1007/s40653-021-00373-x

**Published:** 2021-06-25

**Authors:** Saleh Adel G. A. Al-Tamimi, Gerard Leavey

**Affiliations:** 1 Foxfield, USA; 2 Corelaine, Northern Ireland

**Keywords:** Mental trauma, Realist review, Conflict, Low-middle income country, Community-based interventions

## Abstract

Where low- and middle-income countries have limited economic resources to provide individualized mental health services to people exposed to conflict, community-based interventions may be more appropriate. We aimed to evaluate community level interventions for improving mental health outcomes in Low- and Middle-income countries (LMIC). A realist review of community-based interventions (CBIs) to improve mental health for people in LMIC following conflict. Five databases (Cochrane, PubMed, PsychINFO, Medline, and CINAHL) and a manual search of individual papers. We found 1318 articles, of which 29 were selected. Out of the 29 primary articles, 19 showed successful results, 4 showed mixed results, 1 showed inconclusive results, and 1 showed unsuccessful results. After analyzing the results, we found 3 mechanisms that may influence the effectiveness of these CBIs: the use of lay community members as intervention deliverers, the application of transdiagnostic approaches, and customized outcome assessment tools. Community-based approaches to improve mental health in LMICs are rare and evidence for their effectiveness is limited. Interventions that have a wide scope, train lay mental health workers, and use contextually adapted outcome assessment tools show promise.

## Background

In 2019, an estimated 132 million people in 142 countries were affected by war and violent conflict (WHO, 2019), of whom 87% were living in conflict-affected places, 10% were internally displaced, and 3% were refugees (Guha-Sapir et al., 2015). According to the World Health Organization (van Ommeren, 2019), 20% of people affected by conflict experience a mental disorder and another 10% will go on to develop behaviors that negatively impact their well-being (Murthy & Lakshminarayana, 2006).

Refugees are vulnerable to various mental illnesses (Lebano et al., 2020; Lustig et al., 2004; Bronstein & Montgomery, 2011; Hodes & Vostanis, 2019). While post-traumatic stress reactions, and post-traumatic stress disorder (PTSD) are commonly noted in the conflict literature (van Ommeren, 2019; Kessler et al., 2014), survivors of conflict are also at risk of more common mental disorders such as depression, anxiety and substance use disorders. Moreover, demographic, geographical, and situational risk factors often interact in ways that influence mental health outcomes (Roberts et al., 2009; Hossain et al., 2014; Dimitry, 2012). Women are particularly at risk due to sexual violence (Roberts et al., 2009; Catani et al., 2008). Children, who account for around half of combat-related casualties, are especially vulnerable (Gillies et al., 2016). Additionally, conflict destabilizes institutions that positively impact their intellectual, moral, social, biological, and emotional development, often leading to life-long health problems (Gillies et al., 2016). Identifying mental health symptoms associated with trauma is particularly challenging among children, as their symptom presentation varies wider than in adults (Gillies et al., 2016). More generally, people affected by conflict often experience multiple stressful life events including separation from family members and displacement prior to mental healthcare access (Roberts et al., 2009; Williams & Thompson, 2011). While post-traumatic stress disorder (PTSD) is the most commonly diagnosed disorder among conflict-affected persons, post-conflict stressors including poor nutrition, lack of access to care, a lack of support systems, increased caretaker responsibility, and the ongoing threat of violence all have a compounding effect on mental health problems (Williams & Thompson, 2011). However, there is considerable contextual heterogeneity within conflict situations and experiences that require further investigation about approaches to the conceptualization, diagnosis and treatment of trauma and comorbid symptoms. Low-income countries have a relatively limited mental health workforce. For example, compared with global averages of 3.96 psychiatrists per 100,000 people, many developing countries in Asia and Africa, often have less than 1 per million (Rathod et al., 2017). Addressing the health needs of refugees at the global, national and local levels remains a significant challenge (WHO, 2018).

## Collective Versus Individualist Approaches

An individualism-collectivism framework was initially theorized by Hofstede (1980) in a study involving the multinational corporation IBM across 60 countries and involving over 100,000 participants (Tirodkar et al., 2011). Individualist societies as defined by Hofstede (1980) emphasize the self, autonomy, and independence, in contrast with collectivist societies that emphasize the group, collective identity, solidarity, sharing, and reciprocity. Very generally, Western countries are more strongly characterized as individualistic, compared to Asian or African societies which tend towards collectivism.

Such contrasting attitudes, beliefs and values influence how health interventions are delivered (Karasz & McKinley, 2007) and their acceptability and utilization by individuals, families and communities (Ying, 1990). Understanding how a patient and his/her family see the world and what’s at stake (Kleinman & Benson, 2006) in the context of community are key to understanding the utility and effectiveness of treatment and service delivery (Karasz & McKinley, 2007). Thus, the alignment of interventions with the dominant explanatory model for the required mental health outcome may be crucial in conflict settings, while collectivist approaches to treatment may be more congruent with collectivist cultures such as those in Africa and Asia.

Trauma-focused therapies are generally designed for individuals exposed to traumatic events of war and conflict. Diagnosis is developed within a Western diagnostic framework and treatment is usually provided by qualified and trained mental health professionals. While the treatment modality may differ (e.g. counselling, Eye Movement Desensitization and Reprocessing, psychotropic medication), these interventions are delivered at the individual level with minimal reference to family, group, or community. The delivery of individual trauma-focused treatment may not be feasible in low-middle income countries (LMICs), particularly those affected by conflict and where mental health services are limited and disrupted (Rathod et al., 2017; Burhansstipanov, 1998). In contrast, psychosocial approaches which emphasize the broader social context of trauma and the need for community-level support and healing (Miller & Rasmussen, 2010; Schafer et al., 2014), facilitating recovery by re-building and strengthening social support mechanisms may be more acceptable, impactful and sustainable (Iemmi, 2019).

## Community-Based Interventions

Hypothetically, community-based interventions (CBIs) succeed by promoting mental health, improving social relationships, protecting human rights, and reducing stigma (Kohrt et al., 2018). Community factors can positively influence services delivered in primary healthcare, improve familial/social inclusion, and provide economic benefits unseen in specialty healthcare settings (Kohrt et al., 2018). CBIs are interventions that generally build capacity or aim to facilitate self-efficacy for local groups rather than diagnosing and treating individuals with mental disorders (Williams & Thompson, 2011), and overcome the barriers present in low-middle income countries that can restrict or prohibit access to primary healthcare or specialty settings (Kohrt et al., 2018). These barriers are assumed to be highly context-dependent, operating within the structural and cultural frameworks within any given community, and varying considerably in their scope and delivery (McLeroy et al., 2003).

Four types of CBIs have been identified: community as a setting, community as a target, community as an agent, and community as a resource (Rothman, 1995). Moreover, CBI may contain elements from each and can be delivered in various settings including homes, schools, prisons, community centers, non-government organization facilities, and through text messages, voice calls, video conference calls, and radio (Kohrt et al., 2018). CBIs are implemented by a wide variety of groups, including community-based organizations, non-governmental organizations (NGOs), health ministries and departments, faith-based organizations, professional organizations, and non-profits Salabarría-Peña, 2020). The organizational structure in which CBIs are delivered through differs based on the type of organization, the intervention being delivered, and on contextual factors (McLeroy et al., 2003) and their flexibility in delivery and setting is attractive in low-resource settings (Tyrer & Fazel, 2014).

Because CBIs can be tailored to specific mental health symptoms, or utilize a multimodal design, they can have a practically unlimited scope (Williams & Thompson, 2011; Kohrt et al., 2018). The major barriers posited for the successful implementation of community-based interventions are policy, poverty, psychosocial, and sociocultural (Juengsiragulwit, 2015). The program theory behind community-based interventions is that by involving the community in a way that the community finds acceptable, the community can facilitate the improvement of mental health outcomes in people affected by conflict. Attention to contextual factors may reduce stigma, and improve accessibility, scalability, and treatment adherence (Kohrt et al., 2018; Burhansstipanov, 1998). However, while community-based interventions to tackle mental health problems in conflict settings appear both logical and pragmatic, there is a need to collate evidence on their effectiveness and to examine what mechanisms influence the ways in which these interventions work or do not work.

## Methods

### Aims

To undertake a realist synthesis of community-based psychosocial interventions for people in conflict-related settings.

Realist reviews consider complexity (Pawson et al., 2005; Wong et al., 2013) including cultural, political, and organizational dynamics which may influence the success or otherwise of such interventions. Realist analysis seeks to understand what works for whom, why, and under what circumstances (Wong et al., 2013). Realist approaches attempt to unpack the relationships between context, mechanisms, and outcomes of an intervention (Wong et al., 2013) and their application to different program theories (Lawless et al., 2018; Gwyther et al., 2018). Unlike systematic literature reviews, realist reviews attempt to integrate frameworks and evidence from a wide variety of sources and methodologies (Rycroft-Malone, 2012).

## Search Process

The search included the following databases PUB Med, CINAHL, Psychinfo, Cochrane, and Medline. Google was utilized to retrieve grey literature with the same inclusion and exclusion criteria being applied. The iterative search process included articles from 2010 to 2019 selected on the following inclusion criteria: interventions designed to improve mental health within a low-lower-middle income country (according to World Bank classification), interventions taking place exclusively in countries affected by or currently in conflict, and interventions where the community is involved as an agent, target, setting, or resource. We excluded non-English language publications, study protocols and studies involving pharmaceutical treatment or individual psychotherapy/counselling. Studies including internally displaced people and refugee communities in upper-middle to high income countries were excluded.

The initial search process included the following databases CINAHL, PsychINFO, and PUB Med. Search terms included 1) “community intervention”, “community-based intervention”, “community based program” “community level”, “local”, “community participation”, “community involvement”, “community-involved”, and “community-based treatment”, 2) “mental health”, “mental illness”, “psychological distress”, “mental health symptoms”, “depression”, “anxiety”, “mental health outcomes”, “post-traumatic stress disorder”, “mental wellbeing”, and “post-traumatic stress”, and 3) “low-middle income countries”, “LMIC”, “conflict-affected countries”, “fragile situations”, “fragile countries”, “conflict-affected situations” and “developing countries”. We additionally examined Google Scholar and Google.

## Appraisal and Data Synthesis

Appraisal was conducted based on the relevance of the document in context to the objectives, and on the rigor, or credibility of documents, as per RAMESES protocol (Wong et al., 2013). Qualitative and quantitative data were integrated at the beginning of data synthesis and analysis occurred sequentially. Selected articles went under a repeat full-text review after the data extraction to align data extracted from the articles to context-mechanism-outcome configurations, as well as to find conceptualization of barriers to the implementation. Extracted data were organized by program outcomes and analyzed for mechanisms and contextual factors.

## Results

**Fig. 1:**
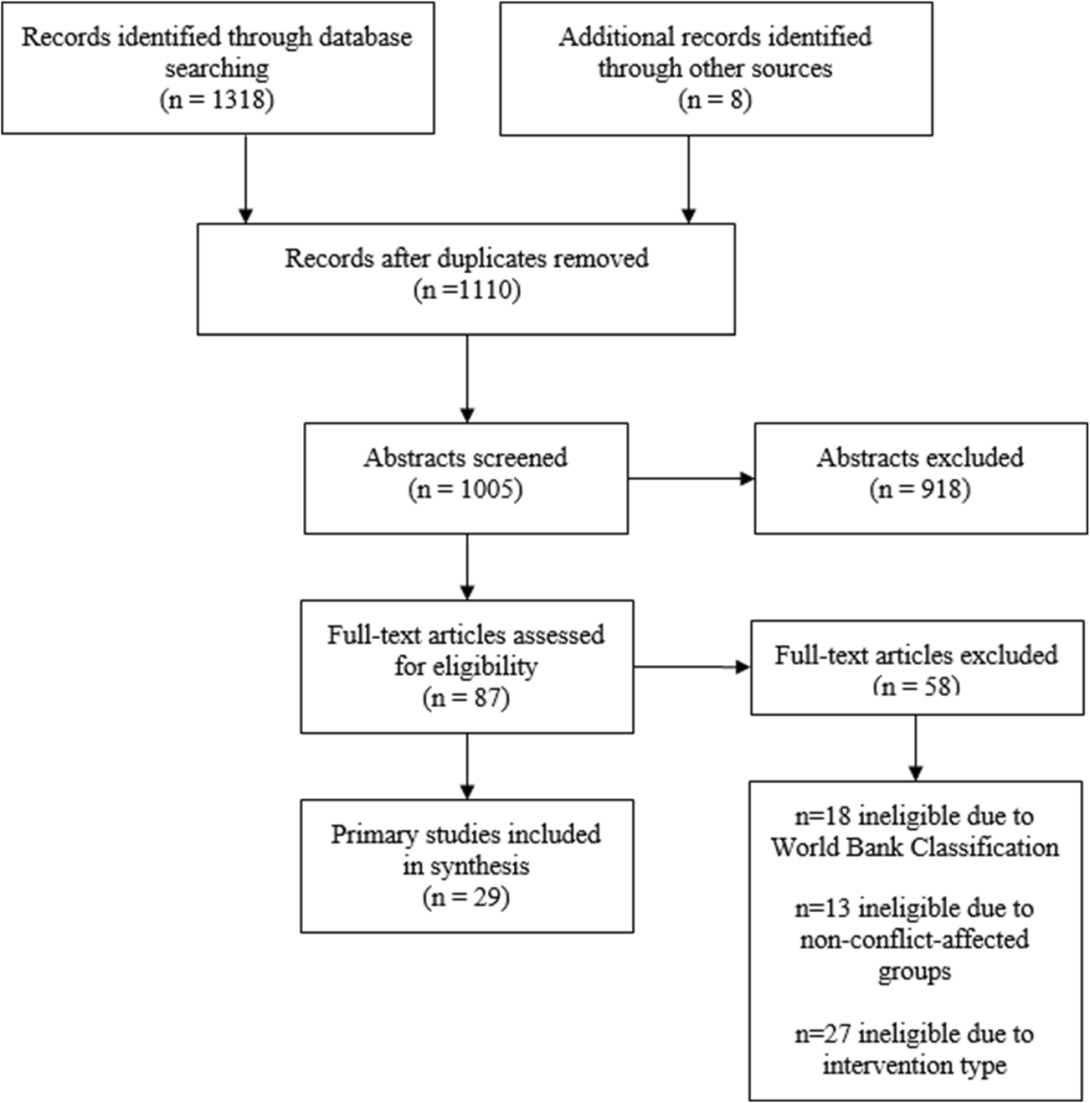
Document flow diagram

We found 1318 articles through database searches and 8 through snowballing, with 218 duplicates. After exclusion criteria was applied, 1005 article abstracts were reviewed, and 87 articles were selected for full-text review based on inclusion criteria. The 29 articles selected as primary studies were selected based on their rigor, and relevance to the research question as per RAMESES protocol, resulting in the exclusion of 58 articles during data extraction (Wong et al., 2013). Out of the 58 studies excluded in the full-text assessment, 18 were removed because they involved a non-LMIC, 13 were removed because the study included non-conflict-affected groups, and 27 were removed because the interventions did not meet the criteria of a community-based intervention. Figure 1 shows the flowchart of studies from identification to inclusion in the iterative search process.

Out of the 29 publications included, 6 were qualitative methods, 14 were RCTs, 3 were literature reviews, 2 were mixed methods, and the remaining 4 were non-RCT quantitative methods. Eight studies took place across multi-country settings, 13 in Africa (12 sub-Saharan countries and 1 Libya), 5 in Asia, and 1 in South America. Two studies involved the community as a target while 5 studies holistically involved in the community (as a resource, setting, target, and facilitator). Most (22/29) studies involved the community as a resource, while 15/29 interventions targeted the whole community. Twenty-one unique interventions were found out of the 29 primary sources. The most prominent intervention was group-delivered trauma-focused cognitive behavioral therapy (TF-CBT) (*n* = 5), followed by narrative exposure therapy (NET) (*n* = 4) and the common elements treatment approach (CETA) (*n* = 2). Two interventions, a school-based intervention and CETA, featured group-delivered TF-CBT as a component of the broader program theory (*n* = 3). Nine focused primarily on children and adolescents, with 7 of those focusing on former combatants, and 1 study focused on former female child combatants. Women were the focus of 3 of the selected studies. Two studies utilized school-based interventions. Timeframes for interventions varied widely, from several days to a year. Table 1 shows the CMO framework synthesized from selected primary studies.

**Table 1:** CMOc framework

Context ➔	Mechanism 1 ➔	Mechanism 2 ➔	Outcome
Lack of human resources	Task shifting	Improved community involvement	Improved cultural acceptability
–	Transdiagnostic elements	–	Improved capacity to treat comorbidities
Lack of high-quality research	Customized outcome assessment tools	Improved community involvement	Improved cultural acceptability
–	–	Context-informed data can be collected	Improved quality of interventions because of tailored interventions from context-informed data
Variety of symptom presentation	Transdiagnostic elements	Improved fluidity of interventions	Treatments can be disseminated further, increasing accessibility
–	Customized outcome assessment tools	Relevant mental health outcomes established	Clearer direction for assessment tool customization in the future; stronger understanding of treatment effectiveness

Stigmatization, construct validity, different symptom expression among groups, and cultural validity of interventions served as cultural barriers to the implementation of community-based interventions (Nickerson et al., 2017). Lack of resources (financial and human), lack of high-quality research on evidence-based community-based interventions, lack of direction for lay community psychosocial workers in handling comorbidity, and lack of incentives for lay community healthcare workers served as material barriers for the implementation of CBIs (Nickerson et al., 2017). CBIs succeed and show effectiveness by accounting for barriers during the implementation process (McLeroy et al., 2003).

## Intervention Effectiveness

CBIs were effective in people with mild or moderate mental health problems. Community based interventions utilizing a trauma informed approach were especially effective for former male child soldiers. However, evidence for the effectiveness CBIs for child soldiers in conflict affected LMICs is limited. This is also the case with girls that have experienced sexual trauma during conflict. Trauma-informed CBIs were generally found to be effective, but the effectiveness of CBIs for former female child combatants is still in question, as the literature almost exclusively focused on former male child combatants. The effectiveness of CBIs for women with mild to moderate mental health problems is mixed.

Community-based interventions were generally effective in improving several mental health related outcomes, including mental well-being, openness to support, and symptoms from post-traumatic stress, anxiety, and depression. However, effect sizes often differed between boys and girls in some interventions, such as TF-CBT. Effect sizes for CBIs with predominantly trauma-focused program theory tended to be significant in more severe cases (Tol et al., 2012). CBIs tended to show effectiveness for general mental well-being outcomes, such as improvements in prosocial behavior and conduct. One review by Kumar and Willman (2016) showed that the combination of psychosocial support and economic empowerment can improve both psychosocial and economic wellbeing. While CBIs with predominantly psychosocial program theories were additionally shown to significantly improve economic security, exclusively economic interventions generally had no significant effect on mental health outcomes (Kumar & Willman, 2016). However, one study of a livestock assets intervention showed a significant moderating effect on mental health symptoms among women who have experienced protracted conflict in D.R. Congo (Glass et al., 2017). This moderating effect could stem from a boost in self-efficacy or self-confidence.

According to Kumar and Willman (2016), the poor outcomes might be due to how financially complex the intervention is. Interventions involving low-interest loans and lines of credit not only showed insignificant results in improving mental health outcomes but instead, reduced mental health wellbeing for people in conflict-affected settings. This may be due to the stress associated with complex financial interventions, especially for target groups that may have little to no formal education due to conflict. The Pigs for Peace intervention studied in Glass et al. (2017) was simple; the NGO gives a pig to a family, and the family is responsible for raising the pig until it can give birth, in which two piglets are given back to the NGO to be given to other women who have experienced conflict in D.R. Congo. These differences highlight the importance of cultural appropriateness in an intervention, and why it is crucial for interventions to be tailored to the specific context.

## Mechanisms

### Customized Tools for Assessing Trauma-Associated Symptoms

Out of the 29 selected studies, 11 studies utilized customized assessment tools either independently, or in combination with more widespread tools including the Harvard Trauma Questionnaire (HTQ), the DSM-5 framework for PTSD, and UCLA PTSD- Reaction Index. The most common tool for studies involving children was the Child Posttraumatic Symptom Scale (CPSS), and the Screen for Anxiety Related Disorders (SCARED). Adapted qualitative methods to ensure outcome assessment tools were culturally considerate, and 1 study utilized an outcome assessment tool designed specifically for women who experienced traumatic symptoms. One study utilized different secondary measures for boys and girls. The most prevalent tool throughout the studies was the HTQ.

The most prominent of the interventions seen in this review, CETA, group-delivered TF-CBT, and NET, all of which were delivered by lay community psychosocial workers, were all successfully implemented by operating around the barriers of limited construct validity, differing symptom expression across cultures, and unclear validity of interventions by customizing psychosocial assessment tools specific to the context in which the intervention is delivered (Kohrt et al., 2010; Murray et al., 2015; Im et al., 2018).

It is not possible to accurately quantify the gap in effectiveness between interventions within the context of this review. However, more prominent tools can still maintain construct validity in assessing the effectiveness of one intervention in multiple contexts if there is some sort of custom, contextually adapted and culturally informed tool that can be designed and implemented alongside the more prominent tool. By observing the changes at baseline, post-intervention, and follow up in both the custom tool and the broader tool, not only can researchers observe effect sizes in community-based interventions for mental health symptoms in LMICs, but also fine-tune and inform future custom tools.

### Trans-Diagnostic Interventions

Most studies (*n* = 16) incorporated transdiagnostic elements of change within their program theories, emphasizing a wholistic approach to symptomology and treatment of trauma-associated mental health symptoms. CETA, Problem Management+, NET, and TF-CBT are examples of such interventions. The comorbid nature of many of the disorders (PTSD, depression, and anxiety) suggest the benefits of such approaches as opposed to a disorder-specific approach. Another element of change that may come into play is the difference in symptom presentation among children compared to adults, men compared to women, the types of trauma experienced between individuals and within communities, and cultural conceptualizations of trauma and mental illness. Transdiagnostic interventions work in congruency with customized assessment tools to allow for CBIs that are flexible enough to be adapted within any context, but consistent enough to be methodologically and clinically vetted.

Transdiagnostic approaches work by identifying the maladaptive psychological processes present in a specific disorder or group of disorders (Gutner et al., 2016). Transdiagnostic approaches are well-established in literature, but mostly in context to eating, anxiety, and depressive disorders (Gutner et al., 2016). Although transdiagnostic approaches show promise and potential in reducing PTSD symptoms, there are few studies supporting the efficacy and effectiveness of any transdiagnostic intervention for PTSD, especially CBIs in conflict affected LMICs with transdiagnostic approaches (Gutner et al., 2016). However, preliminary research shows that interventions, including CBIs that incorporate transdiagnostic elements, show promising results, including to those in low-resource settings (Bolton et al., 2014).

### Task Shifting Approach

Out of the 29 primary sources, 19 utilized task shifting, making it the most frequently observed mechanism. Healthcare task-shifting which seeks to address human resource challenges is a relatively novel concept in global mental health (WHO, 2007). Initially conceptualized in response to the lack of trained healthcare workers in developing countries afflicted with HIV epidemics, it is theorized that by shifting tasks from the normally-highly qualified and highly-educated health workers responsible for delivering interventions to less qualified but adequately trained personnel, more efficient utilization of human resources can be carried out (WHO, 2007). The low costs associated with the implementation of task-shifting approaches have also made these approaches appealing in low-resource settings (Joshi et al., 2014; (Joshi et al., 2014). Task-shifting approaches are well-established in literature, but mostly in context to eating, anxiety, and depressive disorders (Gutner et al., 2016). Barriers to the implementation of task-shifting approaches in CBIs include problems retaining personnel, lack of incentives for personnel, the inability to provide related program materials, resistance from stakeholders and institutions, and acceptability by intervention targets (Joshi et al., 2014).

Trauma-informed psychoeducation delivered to Somali refugees in Kenya with clinically-indicated PTSD by trained youth workers may have been effective due to the utilization of community members as deliverers of trauma-informed psychoeducation, and because the treatment was delivered to severe cases of PTSD (Im et al., 2018). By compensating for language and logistic barriers presented by the program materials, peer-lead group-based recovery was effective. The use of trained peers may have increased the effectiveness of this intervention among Libyans in refugee settlements (Stanford et al., 2013). Problem Management+ may have been successful due to the use of lay community healthcare workers (Rahman et al., 2016).

In community-based interventions for mental health, task shifting includes focusing on administrative duties, implementing interventions, providing care. Task-shifting interventions may be a cost-effective and efficacious method of treating depression (Joshi et al., 2014), but the evidence for this approach is limited, especially in conflict-affected LMICs (Kakuma et al., 2011). In task shifting, the community is utilized as a resource and, to a lesser extent, as a facilitator, as the intervention becomes more culturally acceptable and appropriate if the community has the opportunity to collaborate and provide their insight to the intervention (Joshi et al., 2014.). As the intervention becomes more culturally acceptable and appropriate, the community may also collaborate in the process by providing insight to further tailor the intervention. Koegler et al. (2019) suggested the task shifting approach as a reason why their solidarity group intervention may have been successful.

### Key Findings

CBIs might work in improving mental health outcomes of people living in conflict affected LMICs through at least 3 mechanisms. Task shifting may be utilized to overcome the barrier of low manpower by incorporating the community as intervention deliverers, which in turn, may improve cultural acceptability. By incorporating transdiagnostic elements into CBI program theory, the barrier of varied symptom presentation and conceptualization for trauma-related symptoms may be addressed. In doing so, the adaptability of a CBI in treating different mental health symptoms may improve its effectiveness on a community-wide scale. Customizing outcome assessment tools based on the context in which the intervention is being delivered, and to whom, might address the lack of high-quality research available pertaining to the effectiveness of CBIs. By establishing more relevant mental health outcomes, future researchers may be able to further our understanding of CBI effectiveness while providing a basis for improving customized outcome assessment tools in the future.

### Strengths and Limitations

The methodology of realist reviews come with several disadvantages. Firstly, the results and recommendations provided by realist reviews only support theories pertaining to the CBIs in question. Secondly, realist reviews do not answer which CBIs are the most effective, as well as providing every single effective CBI in the literature. Moreover, it is impossible to clarify the relative contribution of each component. However, we attempted to uncover which successful elements were most prevalent and corroborated. Realist reviews require the ability to make abstract decisions throughout all stages of the realist review process, which bring challenges that are absent in alternative methodologies like systematic literature reviews with meta-analysis, such as an increased risk of errors in the application of realist methodology.

Our study was unable to cover the frequency of delivery, and the duration of individual sessions. These factors may have an impact on the effectiveness and sustainability of the intervention. None of the studies found during the article selection examine the cost-effectiveness or wider financial aspects of community-based interventions, nor the long-term effectiveness of their respective interventions. This is somewhat problematic, as cost serves as a huge barrier towards the implementation of psychosocial interventions, especially in LMICs. There was also a significant absence on the role that gender plays in the etiology of traumatic symptoms in selected articles. Only one article, Bonilla-Escobar et al. (2018), utilized gender-specific outcome screening. The sample size of selected articles, as well as the broad nature of the inclusion and exclusion criteria, illustrates the necessity of high-quality research pertaining to the efficacy of CBIs for mental health in conflict affected LMICs. There was also a notable lack of research pertaining to CBIs for substance abuse in conflict affected LMICs. Finally, the absence of appropriate grey literature that fit the criteria may have been impacted by the dates selected as a part of the exclusion criteria.

This paper contributes to the growing body of literature pertaining to the role that task-shifting, and transdiagnostic elements of change play in CBIs among a demographic that is neglected in many ways, at least financially, academically, and psychologically It is important to consider that there is a significant literature gap pertaining to the effectiveness of psychosocial CBIs. With that said, we believe this paper achieved the established study objectives.

## Conclusion

Only a few community-based interventions for trauma-related symptoms have been studied in the field, and even fewer have been studied in low-resource settings, according to Tyrer and Fazel (2014). For example, interventions directed at refugee children with social and behavioral symptoms aim to improve the ability of children to construct a personal account of their life, express emotion, and interact with others, whereas community-level cognitive behavioral therapy has been indicated as efficacious for reducing post-traumatic symptoms in adults in literature review (Tyrer & Fazel, 2014).

Although the nature of realist reviews does not allow us to make full-truth recommendations, we can still make recommendations for future researchers and community-based intervention implementors based on observable mechanisms that influence the effectiveness of these interventions. Firstly, we recommend that implementers utilize program theories that promote community inclusiveness, whether in the form of community members as intervention deliverers, or as agents of change. Secondly, we recommend that implementers consider the inclusion of transdiagnostic elements into the program theories of community-based interventions. We also recommend conducting high-quality research on the efficacy of CBIs for substance abuse for conflict-affected persons in LMICs. Finally, we recommend that researchers utilize mixed methods approaches that incorporate qualitative methods in customizing outcome assessment tools to be used in combination with more universal outcome assessment tools. Some of the barriers, such as the lack of incentives for lay community healthcare workers, cultural acceptability of interventions, stigmatization, and construct validity were accounted for in some studies, but future research is needed to further understand the role these mechanisms play in the effectiveness of CBIs for symptoms associated with mental trauma in LMIC.
